# Air powder waterjet technology using erythritol or glycine powders in periodontal or peri‐implant prophylaxis and therapy: A consensus report of an expert meeting

**DOI:** 10.1002/cre2.855

**Published:** 2024-02-12

**Authors:** Chun Ching Liu, Neha Dixit, Christian R. Hatz, Tobias M. Janson, Klaus‐Dieter Bastendorf, Georgios N. Belibasakis, Raluca Cosgarea, Ioannis K. Karoussis, Magda Mensi, Jessica O'Neill, Axel Spahr, Andreas Stavropoulos, Patrick R. Schmidlin

**Affiliations:** ^1^ Clinic of Conservative and Preventive Dentistry, Division of Periodontology and Peri‐implant Diseases, Center of Dental Medicine University of Zurich Zurich Switzerland; ^2^ Department of Clinical Affairs and Medical Education Electro Medical Systems SA Nyon Switzerland; ^3^ Dr. Strafela‐Bastendorf Dental Practice Eislingen Germany; ^4^ Department of Dental Medicine, Division of Oral Diseases Karolinska Institutet Stockholm Sweden; ^5^ Department of Periodontology, Cariology and Preventive Dentistry University of Bonn Bonn Germany; ^6^ Department of Periodontology, Faculty of Dentistry National and Kapodistrian University of Athens Athens Greece; ^7^ Section of Periodontics, Department of Surgical Specialities, Radiological Science and Public Health, School of Dentistry University of Brescia Brescia Italy; ^8^ Discipline of Periodontics, School of Dentistry, Faculty of Medicine and Health The University of Sydney Sydney New South Wales Australia; ^9^ Department of Periodontology, Faculty of Odontology University of Malmö Malmö Sweden; ^10^ Division of Conservative Dentistry and Periodontology University Clinic of Dentistry, Medical University of Vienna Vienna Austria

**Keywords:** airflowing, air polishing, peri‐implantitis, periodontitis

## Abstract

**Objectives:**

To attain a collective expert opinion on the use of air powder waterjet technology (APWT) with erythritol and glycine powders in the prophylaxis and therapy of periodontal and peri‐implant diseases.

**Material and Methods:**

In the first step, a modified one‐round online Delphi survey including 44 five‐point Likert scale questions was conducted among a group of 10 expert clinicians and researchers with thorough knowledge and experience in this topic. In the second step, the single questions and the survey results were discussed during a meeting, and consensus statements were formulated, respectively.

**Results:**

An agreement was reached on most items, especially opinions supporting glycine and erythritol powders as favorable with respect to efficiency, safety, and comfort. More scientific evidence is needed to support the improvement in clinical attachment on teeth and implants, especially when APWT with erythritol is used. In addition, APWT needs more long‐term evaluation and studies in terms of microbiome/microbiological effects as well as effects on the inflammatory response on natural teeth and implants, also in light of a guided biofilm therapy concept.

**Conclusions:**

In line with the expert opinions and supported by the evidence, it was concluded that the use of APWT with erythritol and glycine powders in nonsurgical periodontal and peri‐implant therapy and prophylaxis is patient compliant and efficient.

## 1 INTRODUCTION

Periodontal and peri‐implant diseases (i.e., gingivitis/periodontitis and peri‐implant mucositis [PIM]/peri‐implantitis [PIT]) represent inflammatory destructive conditions triggered by microbial biofilms on nonshedding oral surfaces (Renvert et al., 2019; Tonetti et al., 2018).

The main goals of prophylaxis and the first steps of therapy that are indicated in gingivitis/periodontitis and PIM/PI involve a nonsurgical approach focusing on the bacterial hard and soft deposits, that is, removal of calculus, biofilm, and bacterial‐derived toxins, on infected root and implant surfaces, respectively (Berglundh et al., 2018). The so‐called active anti‐infective therapy thereby aims to reduce, ideally eliminate, soft tissue inflammation and suppress disease progression and includes mechanical instrumentation (Sanz et al., 2020). Mechanical instrumentation can be delivered by hand instruments, power‐driven sonic and ultrasonic instruments, as well as lasers and air powder waterjet technology (APWT) (Heitz‐Mayfield & Mombelli, 2014).

Resorbable, minimally traumatic cleaning powders have advanced and gained considerable success in recent years to be integrated clinically as an efficient tool in both periodontal and peri‐implant disease management. These powders have been developed to be less abrasive, soluble, and biocompatible to efficiently remove soft deposits and biofilms from tooth and implant surfaces (Karmakar & Kamath, 2017). The most common are glycine and erythritol powders which have been used in active and supportive periodontal care, as well as in the treatment of PIM/PI. On teeth, APWT with erythritol powder results in comparable improvements regarding probing pocket depths (PPD) and bleeding score reductions as compared to conventional hand and ultrasonic instrumentation in both active and supportive periodontal therapy (APT and SPT) as an adjunct and as a standalone treatment (Abdulbaqi et al., 2022). Similar results have been observed with APWT in the management of PIM and PI, both as a standalone or adjunct therapy in comparison to conventional mechanical debridement methods (Muthukuru et al., 2012; Schwarz, Becker, & Renvert, 2015). The studies included in these reviews (Abdulbaqi et al., 2022; Muthukuru et al., 2012; Schwarz, Becker, & Renvert, 2015) had heterogenous clinical protocols, comparing a plethora of test and control approaches, including machine‐driven (i.e., ultrasonic) debridement, adjunctive local antiseptics, adjunctive local/systemic antibiotics, or laser therapy. Nonetheless, a notable advantage of this technology, especially in view of the supportive periodontal care and peri‐implant maintenance, is enhanced patient comfort and reduced treatment time as reported in multiple systematic reviews (Buhler et al., 2016a; Nascimento et al., 2021; Tan et al., 2022; Zhang et al., 2019).

Previous consensus reports have also concluded that supra‐ and subgingival application of APWT using glycine powders is safe and effective for the removal of biofilms on tooth surfaces and restorative materials (Cobb et al., 2017). In addition, the clinical application of APWT was also as effective for the management of PIM, and statistically significantly higher bleeding on probing reduction (BOP) were reported following nonsurgical PIT treatment compared with classical mechanical debridement (Schwarz, Schmucker, & Becker, 2015). Meanwhile, as additional studies were conducted, a new expert meeting was convened to summarize the latest scientific evidence available and to update the clinical recommendations for the use of APWT in active, nonsurgical, and SPT as well as in peri‐implant maintenance and PIT therapy. For this purpose, scientific questions according to the following nine topics were formulated:

1. Biofilm removal
2. Clinical effectiveness of APWT as an adjunct in periodontal and PIT active therapy
3. Clinical effectiveness of APWT as an adjunct in periodontal and PIT supportive care
4. Safety on tooth substances, dental materials, and soft tissues, and compared to other methods or techniques
5. Patient and clinician comfort compared to other methods or techniques
6. Microbiological effects
7. General safety
8. Clinical statements
9. Study quantity and quality

## 2 MATERIALS AND METHODS

In December 2021, a scientific board was constituted. The group consisted of 12 experienced international clinicians and researchers in the field of periodontitis and peri‐implant disease. In the first round, the panel was asked to take part in an anonymous one‐round modified Delphi survey. This method works on the principle that a consensus or agreement can be reached by using a structured group of experts. The Delphi method normally consists of multiple question/answer/feedback rounds, which are performed over multiple rounds, until consensus is reached (Ab Latif et al., 2016; Linstone & Turoff, 2002). In the present work, a simplified format was used in preparation for a physical expert meeting, which was held during the EFP Europerio 10 2022 Congress in Copenhagen, Denmark.

The survey was based on an umbrella review project, which was performed to evaluate the actual scientific evidence on APWT in periodontal and PI therapy (Hatz et al., 2022). The survey consisted of nine thematic blocks and respective answers were given in a 5‐point Likert scale with values from +2 to −2, with the zero value for “Neutral” in each case dividing responses between positive and negative assessments. For the descriptive data presentation, the respective median values were provided. To obtain a statistical estimation of the actual values around the zero point, a Wilcoxon test was applied to a sample with a significance level of 0.01 and a test value of 0 (DATAtab Team, 2022, DATAtab: Online statistics calculator. DATAtab e.U. Graz, Austria. https://datatab.net).

The results of the online survey were presented during the meeting and discussed. Votes were cast using anonymous software as part of a moderated, nominal group process. A simple voting system (yes = agree; no = disagree) was applied. The results expressed as percentage of the “yes” and “no” votes were assigned to the following categories (consensus strength):

– Strong agreement agreement of >95% of the participants
– Majority agreement agreement of 75%–95% of the participants
– Agreement agreement of 50%–75% of the participants
– No agreement agreement of <50% of the participants

## 3 RESULTS

In the following short sections, the results of the different evaluated topics of the two rounds are presented. The results of the first‐round survey, with, for example, a Likert scale ranging from very efficient (score 2) over neutral (score 0) to very inefficient (score −2), are described by median values and respective *p*‐values.

The results of the meeting are reflected as expert statements reflecting the nine topics.

### 3.1 Biofilm removal

#### 3.1.1 Results of the first‐round online survey

Results of the first‐round online survey for biofilm removal (Table 1).

**Table 1:** Results of the first‐round survey regarding biofilm removal (very efficient: 2; neutral: 0; very inefficient: −2).

**Biofilm removal**	**At teeth**	**At implants**
Supragingivally	**2** (*p* < .001)	
Marginally (epigingivally)	**2** (*p* < .001)	**2** (*p* < .001)
Subgingivally, flat surfaces	**1** (*p* < .001)	**1** (*p* = .001)
Subgingivally, furcations	(*p* = .001)	

*Note*: Median values are provided in bold numbers (*p*‐values in brackets; statistically significant values indicate that the responses differ from zero and represent therefore a respective opinion).

#### 3.1.2 Conclusions of the expert group

a. Supragingivally, APWT is efficient in removing biofilms at teeth and implants
  – Strong agreement (100%)

b. Subgingivally, APWT is efficient in removing biofilms from teeth
  o Flat surfaces: Strong agreement (100%)
  o Furcation areas: Strong agreement (100%), depending on access
c. Submucosally, APWT is efficient in removing biofilms at implants
  – Majority agreement (73% agreement), depending on access

### 3.2 Effect on clinical parameters as an adjunct during active therapy

#### 3.2.1 Results of the first‐round online survey

Results of the first‐round online survey for effect on clinical parameters as an adjunct during active therapy (Table 2).

**Table 2:** Results of the first‐round survey clinical efficiency as an adjunct therapy during active therapy (high: 2; neutral: 0; very low: −2).

	At teeth	At implants
Nonsurgically		
Bleeding on probing (BoP)	**1** (*p* = .008)	**1** (*p* = .001)
Probing pocket depths (PPD)	**1** (*p* = .001)	**1** (*p* = .004)
Clinical attachment level (CAL)	**1** (*p* = .001)	**0** (*p* = .157)
Surgically		
BoP	**0** (*p* = .008)	**1** (*p* = .004)
PPD	**0** (*p* = .025)	**0** (*p* = .014)
CAL	**0** (*p* = .015)	**0** (*p* = .063)

*Note*: Median values are provided in bold numbers (*p*‐values in brackets; statistically significant values indicate that the responses differ from zero and represent therefore a respective opinion).

#### 3.2.2 Conclusions of the expert group

a. During APT, APWT is effective in improving
  – BoP: Strong agreement (100%)
  – PPD: Strong agreement (82%)
  – Clinical attachment level (CAL): Borderline agreement (50%)

b. During active peri‐implant therapy, APWT is effective in improving
  – BoP: Strong agreement (92%)
  – PPD: Borderline majority agreement (75%)
  – CAL: No agreement (42%)

### 3.3 Effect on clinical parameters as an adjunct during supportive care

#### 3.3.1 Results of the first‐round online survey

Results of the first‐round online survey for effect on clinical parameters as an adjunct during supportive care (Table 3).

**Table 3:** Results of the first‐round survey clinical efficiency as an adjunct therapy during supportive therapy (high: 2; neutral: 0; very low: −2).

	At teeth	At implants
Nonsurgically		
Bleeding on probing (BoP)	**2** (*p* = .001)	**1** (*p* = .001)
Probing pocket depths (PPD)	**1** (*p* = .003)	**1** (*p* = .009)
Clinical attachment level (CAL)	**1** (*p* = .004)	**0** (*p* = .059)

*Note*: Median values are provided in bold numbers (*p*‐values in brackets; statistically significant values indicate that the responses differ from zero and represent therefore a respective opinion).

#### 3.3.2 Conclusions of the expert group

a. During SPT, APWT is effective in improving
  – BoP: Strong agreement (100%)
  – PPD: Majority agreement (92%)
  – CAL: Agreement (58%)

b. During peri‐implant maintenance, APWT is effective in improving
  – BoP: Strong agreement (100%)
  – PPD: Borderline majority agreement (75%)
  – CAL: No agreement (25%)

### 3.4 Safety on teeth, restorative materials, and soft tissues

#### 3.4.1 Results of the first‐round online survey

Results of the first‐round online survey for safety on teeth, restorative materials, and soft tissues (Table 4).

**Table 4:** Results of the first‐round survey regarding the safety on dental hard tissues, dental materials, and soft periodontal/peri‐implant tissues (very safe: 2; neutral: 0; very unsafe: −2).

Enamel	2 (*p* = .001)
Dentin	**1** (*p* = .001)
Cementum	**1** (*p* = .001)
Unfilled resins	**1** (*p* = .002)
Provisional materials	**1** (*p* = .001)
Composite resin materials	**1** (*p* = .001)
Ceramic materials	**1** (*p* = .001)
Luting materials/interfaces	**1** (*p* = .001)
Gingiva (around teeth)	**2** (*p* = .001)
Mucosa (around implants)	**1** (*p* = .001)
Tongue	**1** (*p* = .002)
Palate	**1** (*p* = .001)

*Note*: Median values are provided in bold numbers (*p*‐values in brackets; statistically significant values indicate that the responses differ from zero and represent therefore a respective opinion).

#### 3.4.2 Conclusions of the expert group

a. APWT is safe on dental hard tissues, that is, enamel, dentin, and cementum: Strong agreement (100%)
b. APWT is safe on different dental materials: Strong agreement (100%)
c. APWT is safe on periodontal and peri‐implant soft tissues as well as on other oral soft tissues: Strong agreement (100%)
d. APWT is safe for soft tissues as compared to hand instruments, ultrasonic devices, and rubber cups: Strong agreement (100%)

### 3.5 Patient and clinician comfort as compared to other methods

#### 3.5.1 Results of the first‐round online survey

Results of the first‐round online survey for patient and clinician comfort as compared to other methods (Table 5).

**Table 5:** Results of the first‐round survey regarding patient and clinician comfort as compared to other methods (very comfortable: 2; neutral: 0; very uncomfortable: −2).

	Patients	Clinicians
Hand instruments	**2** (*p* = .001)	**2** (*p* = .001)
Ultrasonic devices	**2** (*p* = .001)	**2** (*p* = .001)
Rubber cup	**1** (*p* = .002)	**1** (*p* = .001)

*Note*: Median values are provided in bold numbers (*p*‐values in brackets; statistically significant values indicate that the responses differ from zero and represent therefore a respective opinion).

#### 3.5.2 Conclusions of the expert group

a. APWT is comfortable for patients when compared to hand instruments, ultrasonic instruments, and rubber cups: Strong agreement (100%)
b. The application of APWT is comfortable for clinicians as compared to hand instruments, ultrasonic devices, and rubber cups for biofilm removal: Strong agreement (100%)

### 3.6 Microbiological effects

#### 3.6.1 Results of the first‐round online survey

Results of the first‐round online survey for microbiological effects (Table 6).

**Table 6:** Results of the first‐round survey regarding the microbiological effects in the first 6 months and after (very high: 2; neutral: 0; very low: −2).

	At teeth	At implants
First 6 months	**1** (*p* = .004)	**1** (*p* = .008)
More than 6 months	**0** (*p* = 1)	**0** (*p* = 1)

*Note*: Median values are provided in bold numbers (*p*‐values in brackets; statistically significant values indicate that the responses differ from zero and represent therefore a respective opinion).

#### 3.6.2 Conclusions of the expert group

a. APWT has a positive microbiological effect on tooth and implant surfaces in the short term (≤180 days): Strong agreement (100%).
b. APWT needs more long‐term evaluation and studies in terms of microbiological effects on natural teeth and implants: Majority agreement (86%).

### 3.7 Safety and aerosol production

#### 3.7.1 Results of the first‐round online survey

Results of the first‐round online survey for safety and aerosol production (Table 7).

**Table 7:** Results of the first‐round survey regarding safety and aerosol production (very safe: 2; neutral: 0; very unsafe: −2).

High‐vacuum or ‐speed suction	**1** (*p* = .001)
Saliva ejector or low‐volume evacuator	**0** (*p* = .448)
Two‐handed dentistry (i.e., without a dental assistant)	**1** (*p* = .048)
Four‐handed dentistry (i.e., with the aid of a dental assistant)	**1** (*p* = .001)

*Note*: Median values are provided in bold numbers (*p*‐values in brackets; statistically significant values indicate that the responses differ from zero and represent therefore a respective opinion).

#### 3.7.2 Conclusions of the expert group

a. APWT is less safe when used with a saliva ejector or low‐volume evacuator in regard to aerosol production. Therefore, it is recommended to be used with a high vacuum suction: Strong agreement (100%).
b. APWT is safe when used in four‐handed dentistry and two‐handed dentistry with regard to aerosol production: Strong agreement (100%).

### 3.8 Clinical statements

#### 3.8.1 Results of the first‐round online survey

Results of the first‐round online survey for clinical statements (Tables 8a and 8b).

**Table 8a:** Results of the first‐round survey regarding some clinical statements (strongly agree: 2; neutral: 0; strongly disagree: −2).

	**At teeth**	**At implants**
The removal of calculus is less important than the eradication of soft debris or biofilm	**−1** (*p* = .123)	**−1** (*p* = .04)
Erythritol powder cleans more efficiently and maintains better surface integrity than any conventional technology	**1** (*p* = .015)	**1** (*p* = .01)
Erythritol powder can be used as an adjunct during active periodontal therapy OR even as an alternative to conventional mechanical debridement (i.e. stand‐alone therapy)	**0** (*p* = .963)	**0** (*p* = .1)
Air powder waterjet technology has no or only minute adverse effects on teeth and implants	**1** (*p* = .004)	**1** (*p* = .004)

*Note*: Median values are provided in bold numbers (*p*‐values in brackets; statistically significant values indicate that the responses differ from zero and represent therefore a respective opinion).

**Table 8b:** Results of the first‐round survey regarding the risk of silicosis using two resorbable powders (strongly agree: 2; neutral: 0; strongly disagree: −2).

	**Glycine**	**Erythritol**
Fine‐sized powders with amorphous silica cause NO silicosis as they are completely soluble and resorbable	**1** (*p* = .009)	**1** (*p* = .009)

*Note*: Median values are provided in bold numbers (*p*‐values in brackets; statistically significant values indicate that the responses differ from zero and represent therefore a respective opinion).

#### 3.8.2 Conclusions of the expert group

a. The removal of calculus is as important as the eradication of soft debris or biofilm on teeth and implants: Majority agreement (79% and 93% for teeth and implants, respectively)
b. Erythritol powder cleans efficiently like other technologies and maintains better surface integrity on teeth and implants: Strong agreement (100%)
c. APWT has no or only minute adverse effects on implants: Strong agreement (100%)
d. Erythritol and glycine powder do not cause silicosis as they are completely soluble and biocompatible (100%)

### 3.9 Study quantity and quality

#### 3.9.1 Results of the first‐round online survey

Results of the first‐round online survey for study quantity and quality (Table 9).

**Table 9:** Results of the first‐round survey regarding study quantity and quality regarding the evaluated topics (exhaustive: 2; neutral: 0; very poor: −2).

	**Quantity**	**Quality**
Clinical efficiency	**1** (*p* = .02)	**1** (*p* = .005)
Safety	**0** (*p* = .014)	**0** (*p* = .096)
Comfort	**1** (*p* = .031)	**1** (*p* = .087)
Microbiology	**0** (*p* = .366)	**0** (*p* = .564)
Aerosol management	**−1** (*p* = .083)	**−1** (*p* = .02)

*Note*: Median values are provided in bold numbers (*p*‐values in brackets; statistically significant values indicate that the responses differ from zero and represent therefore a respective opinion).

#### 3.9.2 Conclusions of the expert group

 a) *Study quantity*

a. Study quantity concerning clinical efficiency and comfort is sufficient: Majority agreement (79% and 85%, respectively)
b. Study quantity concerning safety and microbiology is rather sufficient: Agreement (57% and 64%, respectively)
c. Study quantity concerning aerosol is still insufficient: Strong agreement (100%)

 b) *Study quality*

a. Study quality concerning safety is sufficient: No agreement (50%)
b. Study quantity concerning safety is rather sufficient: A majority agreement (79%)

## 4 DISCUSSION

This expert meeting aimed to summarize and pinpoint the current opinions concerning APWT in nonsurgical periodontal and peri‐implant treatment and supportive care with erythritol and glycine powders.

Professional mechanical plaque removal (PMPR) which consists of the removal of supragingival dental biofilm and calcified deposits is the basis for plaque‐induced periodontal treatment and also in the supportive periodontal care phase. This in combination with the subgingival instrumentation is necessary to reduce gingival inflammation and to treat diseased sites (Sanz et al., 2020). A recent systematic review on mechanical decontamination of dental implant reported “air abrasive” systems as a method for nonsurgical PIT treatment. It could be shown that BOP was reduced 3–6 months after nonsurgical approach (Cosgarea et al., 2023).

Consensus was reached on most statements. Soluble cleaning powders used for APWT are favorable, especially with respect to efficiency, safety, and comfort. However, the group identified that for Erythritol powder, there is a need to conduct more clinical studies with respect to improvement in clinical attachment on teeth and implants. In addition, more long‐term studies in terms of microbiome/microbiological effects as well as effects on the inflammatory response are required especially in light of a systematic clinical concept like, for instance, guided biofilm therapy.

Since the last consensus meetings were conducted, multiple new RCTs and systematic reviews have been published in the field of AWPT in both, the treatment of periodontitis and peri‐implant diseases. Therefore, due to new powder developments and technological improvements, an update was desirable. Besides known advantages of safety and patient comfort, user‐specific aspects were assessed, as well as the impact on hard and soft tissues, restorations and implants play a crucial role in the implementation of the powders in general. In addition, the safety and comfort of patients as well as the clinicians were assessed.

The present expert meeting certainly harbored some methodological shortcomings, such as the limited number of participants with a predominant representation from Europe. In addition, due to organizational restrictions and limited time, only a modified one‐round Delphi survey was conducted. However, the obtained results seem to be underlined by the existing literature (Hatz et al., 2022).

While the EFP guideline for periodontitis by Sanz et al. (2020) does not specifically address air polishing as a stand‐alone or adjunctive method in the treatment of Stage I–III periodontal therapy, numerous systematic reviews and randomized controlled trials provide evidence supporting the efficacy of APWT in both active and SPT (Hatz et al., 2022; Nascimento et al., 2021; Tan et al., 2022).

In accordance with the EFP guideline for PIT (Herrera et al., 2023), PMPR using APWT with glycine or erythritol powders either individually or in combination, can be used in patients treated for PIT to mitigate the risk of disease recurrence (Herrera et al., 2023). Additionally, for patients with PIM, APWT may be considered a solitary approach to PMPR (Herrera et al., 2023).

Focusing on the clinical perspective, there is no doubt that effective biofilm removal on both natural teeth and implants is of utmost importance and a strong agreement could be achieved on this central aspect. APWT is considered very efficient in this respect on both teeth and implants, supra‐ as well as subgingivally, but is also strongly dependent on the accessibility of the targeted site (Bennani et al., 2015; Discepoli et al., 2022; Mensi et al., 2020; Ronay et al., 2017; Wenzler et al., 2021). The strong agreement considering the adjunctive use of APWT in nonsurgical periodontal therapy in terms of improvements of clinical parameters especially with respect to BOP and PPD is also in accordance with recent systematic reviews (Abdulbaqi et al., 2022; Nascimento et al., 2021). In terms of CAL gain, no agreement for the benefit of adjunctive use of APWT in active periodontal and peri‐implant therapy could be achieved. This is again in agreement with the literature (Nascimento et al., 2021). So far, only one meta‐analysis showed statistically significant improvements in CAL using APWT in comparison to conventional SRP in teeth (Abdulbaqi et al., 2022).

Moreover, regarding supportive periodontal and peri‐implant care, the statements reached by the experts are in agreement with the literature showing that APWT used as a stand‐alone or as an adjunct seems to be just as effective, in terms of improving PPD, CAL, and BOP, as conventional mechanical debridement alone (Abdulbaqi et al., 2022; Ng et al., 2018; Tan et al., 2022; Zhu et al., 2021). Although APWT has been shown to yield a similar microbial load reduction as the conventional mechanical approach, substantial and clinical relevant improvements are still to be shown, especially in the long‐term microbiological evaluations (≥180 days) (Hentenaar et al., 2022; Kargas et al., 2015; Lu et al., 2018).

The effect of APWT with erythritol–chlorhexidine powder on the subgingival microbiome was recently investigated in a clinical trial (Mensi et al., 2021). It was shown that in the test group, the subgingival use of erythritol–chlorhexidine powder adjunctively to scaling and root planning has a beneficial effect on the reduction of the pathogenic specimens (e.g., *Filifactor alocis, Tannerella forsythia, and Treponema denticola*). It seems to promote a shift toward a more eubiotic condition. This was shown by the increased detection of health‐related specimens (*Abiotropha defective, Capnocytophaga sputigena*, and *Lautropia mirabilis*).

Clinical studies and reviews also reported patient‐related outcomes such as better patient comfort and less treatment pain with the use of APWT. Reports regarding clinician comfort and safety were also in keeping with the consensus (Bühler et al., 2016b; Fu et al., 2021; Muller et al., 2014; Nascimento et al., 2021; Ulvik et al., 2021).

The group was in full agreement that AWPT and the powders utilized are safe on hard and soft oral tissues, restorative materials, and implants as shown in the literature (Arefnia et al., 2021; Barnes et al., 2014; Petersilka et al., 2018). Both erythritol and glycine powder have been shown to be low abrasive, soluble, and biocompatible (De Cock et al., 2016; Hagi et al., 2015; Petersilka et al., 2003).

Regarding the topic of aerosol production after APWT application, a recent study by Mensi et al. was published just after the expert meeting, comparing several dental treatment methods including APWT, ultrasonic instrumentation, rubber cup polishing, and cavity preparations. The study concluded that professional oral hygiene procedures do not result in a higher bacterial count or air contamination produced by aerosols than baseline. In comparison, cavity preparation with turbine handpieces produced significantly higher bacterial counts after 10 min of treatment (Mensi et al., 2022).

Following a review of the most recent literature, a couple of case reports have emerged concerning the occurrence of subcutaneous emphysema (Basetti et al., 2014) and pneumocephalus (Bruckmann et al., 2022) associated with the application of glycine‐ and erythritol‐based powders in air powder water jet technology in the management of peri‐implant complications. Practitioners need to be mindful of the potential risk of iatrogenic emphysema. Rapid diagnosis of subcutaneous emphysema is crucial; treatment modality may depend on the severity of the condition and the overall health of the patients.

Despite a good to excellent agreement on most addressed aspects as delineated above, there remains a need for further studies on the effect of APWT on CAL improvement at teeth and implants as well as safety aspects. Furthermore, more long‐term investigations of the microbiome/microbiological and anti‐inflammatory effects of the APWT application around teeth, implants, and the oral cavity are required.

Additional consensus was reached with respect to terminology. The panel discussed and agreed to use the term “air flowing” to replace “air polishing,” or “air abrasion.”

There have been a lot of discussions around the terminologies “air abrasion” and “air polishing” and the novel terminology “air flowing” has already been quoted in a recent publication (Donnet et al., 2021). The term “air abrasion” was used in the past to remove carious lesions with aluminum‐based hard powders. However, this term should not be used for biofilm removal during professional prophylaxis, where oral hard and soft tissues should not be damaged. “air polishing” is currently the term used for biofilm removal using powders like erythritol, glycine sodium bicarbonate, calcium carbonate, and so on. However, this term could be misleading since polishing may communicate the creation of a new and smoother surface. Thus, “air flowing” should be considered as novel terminology, which follows the same principles of “air polishing” (air, powder, and water) but using a specific device/technology that provides a continuous, consistent, and regulated flow of specifically developed and indicated resorbable powders (i.e., erythritol powder particle size 14 µm). Therefore, the term “Air Flowing” was agreed upon by the expert panel for future use especially with “APWT” when a specific device with erythritol‐based powders is utilized.

## 5 CONCLUSIONS

The expert consensus conference reported that the use of APWT in nonsurgical periodontal or peri‐implant prophylaxis and therapy with Erythritol and Glycine powders is effective, comfortable, and safe on natural teeth and implants.

Implications and recommendations for future studies:

1. Further data are needed to support CAL improvement around teeth and implants
2. Data on long‐term investigations regarding the microbiome/microbiological and anti‐inflammatory effects on teeth and implants are required
3. Further studies should be generated regarding aerosol production
4. High‐quality studies addressing all safety aspects are recommended

## AUTHOR CONTRIBUTIONS

**Chun Ching Liu**: Methodology; data curation; investigation; project administration; visualization, writing—original draft preparation; writing—review and editing. **Neha Dixit**: Conceptualization; methodology; data curation; investigation; project administration; resources; writing—original draft preparation; writing—review and editing. **Christian R. Hatz**: Data curation; visualization; writing‐‐original draft preparation; writing—review and editing. **Tobias M. Janson**: Data curation; visualization; writing—original draft preparation; writing—review and editing. **Klaus‐Dieter Bastendorf**: Conceptualization; methodology; investigation; project administration; resources; writing—review and editing**. Georgios N. Belibasakis, Raluca Cosgarea, Ioannis K. Karoussis, Magda Mensi, Jessica O'Neill, Axel Spahr, Andreas Stavropoulos**: Investigation; writing—review and editing. **Patrick R. Schmidlin**: Conceptualization; methodology; formal analysis; software; investigation; supervision; visualization; writing—review and editing.

## CONFLICT OF INTEREST STATEMENT

Chun Ching Liu was reimbursed for travel expenses for the expert meeting. Neha Dixit is the Global lead, Clinical Affairs and Medical Education at EMS. Klaus‐Dieter Bastendorf is a member of the Advisory Board of EMS and Scientific Board of ADIC, and receives consulting fees and support for attending meetings and/or travel. Raluca Cosgarea, Jessica O'Neill, Axel Spahr, and Patrick R. Schmidlin receive Honoraria for lectures and hands‐on workshops and support for attending meetings and/or travel. Ioannis K. Karoussis and Magda Mensi receive Honoraria for lectures and hands‐on workshops. The other authors declare no conflict of interest.

## Data Availability

The data that support the findings of this study are available from the corresponding author upon reasonable request.
